# Pulmonary histoplasmosis presenting as chronic productive cough, fever, and massive unilateral consolidation in a 15-year-old immune-competent boy: a case report

**DOI:** 10.1186/1752-1947-5-374

**Published:** 2011-08-15

**Authors:** Rodrick Kabangila, Kilonzo Semvua, Peter Rambau, Kahima Jackson, Stephen E Mshana, Hyasinta Jaka, Robert N Peck

**Affiliations:** 1 Department of Medicine, Weill Bugando University College of Health Sciences, Mwanza, Tanzania; 2 Department of Medicine, Bugando Medical Centre, Mwanza, Tanzania; 3 Department of Pathology, Weill Bugando University College of Health Sciences, Mwanza, Tanzania; 4 Department of Microbiology, Weill Bugando University College of Health Sciences, Mwanza, Tanzania; 5 Department of Medicine, Weill Cornell Medical College, 440 East 69th Street, New York, NY 10065, USA

**Keywords:** histoplasmosis, immune-competent, consolidation, acid-fast bacilli, periodic acid-Schiff staining

## Abstract

**Introduction:**

Severe histoplasmosis is known to be among the AIDS-defining opportunistic infections affecting patients with very low CD4 cell counts in histoplasmosis-endemic areas. *Histoplasma capsulatum *var. *duboisii *is common in West and Central Africa, where it occurs in both HIV/AIDS and non-HIV patients. Few cases of life-threatening histoplasmosis in immune-competent individuals have been reported worldwide.

**Case report:**

We describe a case of pulmonary histoplasmosis diagnosed on the basis of autopsy and histological investigations. A 15-year old East African immune-competent boy with a history of smear-positive tuberculosis and a two-year history of rock cutting presented to our hospital with chronic productive cough, fever, and massive unilateral consolidation. At the time of presentation to our hospital, this patient was empirically treated for recurrent tuberculosis without success, and he died on the seventh day after admission. The autopsy revealed a huge granulomatous lesion with caseation, but no acid-fast bacilli were detected on several Ziehl-Neelsen stains. However, periodic acid-Schiff staining was positive, and the histological examination revealed features suggestive of *Histoplasma *yeast cells.

**Conclusion:**

Severe pulmonary histoplasmosis should be considered in evaluating immune-competent patients with risk factors for the disease who present with pulmonary symptoms mimicking tuberculosis.

## Introduction

Globally, histoplasmosis is known to be more frequent in the United States than elsewhere, but it is not uncommon in other parts of the world, including Africa [1,2]. *Histoplasma capsulatum *var. *duboisii *is common in West and Central Africa, where it occurs in both HIV-positive and HIV-negative patients [3,4]. Despite that fact that life-threatening histoplasmosis (chronic, progressive, or disseminated disease) is reported more commonly among immunocompromised and very elderly patients, it has been shown that up to 20% of severe illnesses result from heavy inoculums in healthy and young people [3-5]. In addition, patients with underlying lung disease may develop chronic pulmonary histoplasmosis with clinical and radiographic findings that resemble those seen in reactivation tuberculosis (TB) [6]. Without treatment, the illness is progressive, causing loss of pulmonary function in most patients and death in about half the patients [6,7]. The prevalence of histoplasmosis has not been well established in Africa among HIV-negative patients, which could be due to misdiagnosis in this part of the world because of physicians' lack of awareness [8].

We report the presentation, misdiagnosis, and autopsy findings of a 15-year-old immune-competent boy who presented to our facility with a chronic productive cough, fever, and massive unilateral consolidation. In this report, we also discuss the specific challenges related to the diagnosis and treatment of pulmonary histoplasmosis in resource-limited settings.

## Case presentation

A 15-year-old East African boy was referred to our hospital with a diagnosis of recurrent TB. Upon review, the patient was found to have a history of productive cough and intermittent low-grade evening fevers for one year and shortness of breath for one week prior to presentation. One year and five months prior to his presentation to our hospital, he had presented to a peripheral hospital with similar symptoms and had been diagnosed with sputum acid-fast bacilli (AFB) and smear-positive TB. He had been treated with anti-TB medications, which brought him only mild relief. Two months after completing anti-TB therapy, his cough and fevers worsened, for which a re-treatment regimen of anti-TB medications, including two months of intramuscular streptomycin, were started on the basis of clinical findings. The sputum examination for AFB had not been repeated. Four months into the course of his second course of anti-TB therapy, the patient stopped taking his medications because he reportedly experienced only mild relief of his symptoms. Repeat Ziehl-Neelsen staining performed two months prior to his admission to our institution was negative for AFB, and no more drugs were given. One week prior to his admission to our hospital, the patient developed progressively worsening shortness of breath, which was even worse while he was lying flat and was associated with dull left-sided chest pain.

His social history was significant for crushing stones (rock cutting) for two years, but he had no history of smoking or working in the mining industry. He had no history of TB or other lung disease in his family.

His physical examination revealed that he was fully conscious, afebrile, slightly wasted, and dyspneic. His oxygen saturation level was 96% on room air, and his other vital signs were normal. His respiratory examination revealed a respiratory rate of 25 cycles/minute and chest bulging on his left side, but his trachea was centrally located. Other findings on the left side included decreased chest expansion, increased tactile vocal fremitus, dull percussion note, and bronchial breath sounds. The rest of the respiratory examination was unremarkable.

**Figure 1:**
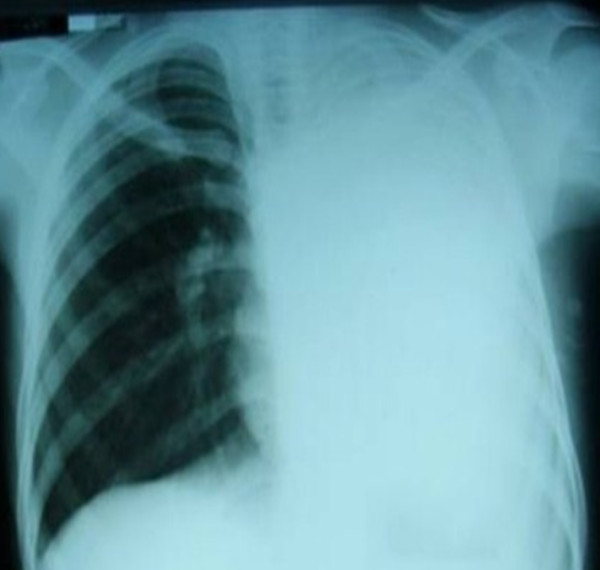
**Chest radiograph showing massive consolidation of the left lung**.

The patient was admitted to the medical ward with a diagnosis of recurrent TB with massive left-sided consolidation. A complete blood count revealed a white blood cell count of 8.7 cells/mm^3 ^with a differential of 69% neutrophils, 23% lymphocytes, 6% monocytes, and 1% eosinophils. His hemoglobin level was 10.6 g/dL, and his platelet count was 504 cells/mm^3^. His erythrocyte sedimentation rate was 20 mm/hour. His renal and liver function tests were within normal ranges. A rapid test for HIV was performed and was negative. Sputum tests for Gram staining and Ziehl-Neelsen staining were both negative. A chest radiograph revealed features of huge left-sided consolidation with complete opacification of the left hemithorax (Figure 1). His chest ultrasound revealed that the left lung was completely consolidated. There was no pleural or pericardial effusion, and his right lung was completely normal. Following these examinations, the patient was empirically re-started on the retreatment regimen for recurrent TB, and the cardiothoracic surgery team was consulted about performing a lung biopsy. The cardiothoracic surgery team felt that the patient was too unstable for surgery and requested that medical management be continued until the patient's condition improved.

On hospital day 3, the patient's condition started deteriorating, with worsening shortness of breath, wheezing, and hypoxia (oxygen saturation 82% to 90% on room air). He was admitted to the intensive care unit, where his anti-TB medications were continued, together with oxygen therapy. On hospital day 7, the patient died before any further intervention could be performed.

**Figure 2:**
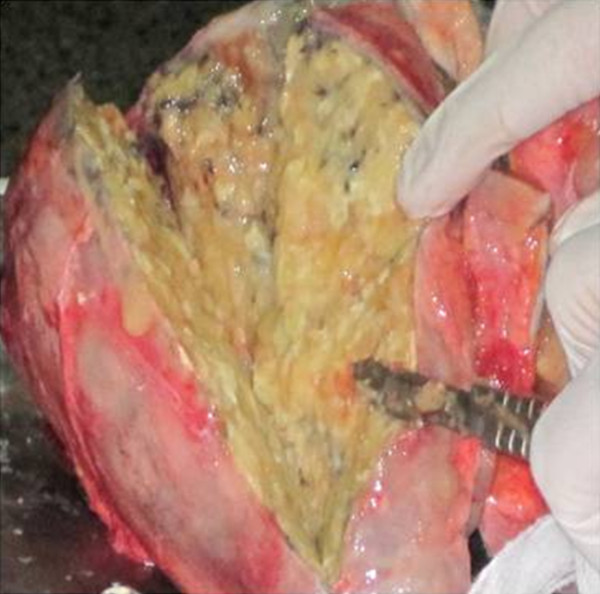
**Gross morphology of the left lung observed at autopsy**. Note the necrotic tissue and caseating granulomas.

**Figure 3:**
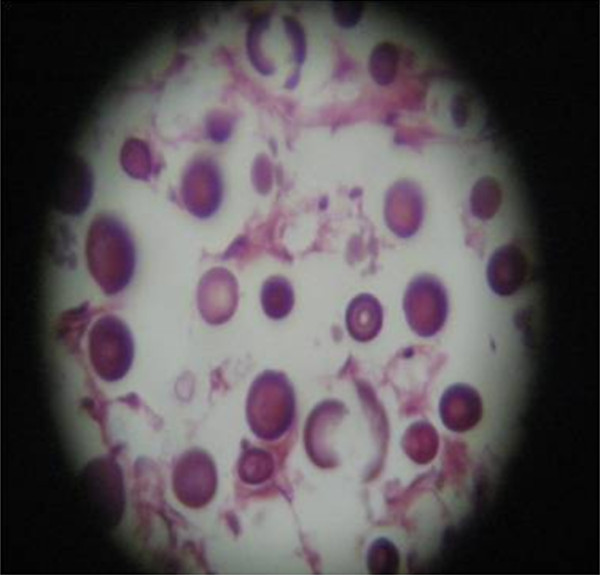
**Hematoxylin and eosin staining showing yeast cells consistent with *Histoplasma *spp**.

A post-mortem examination was performed by the hospital's pathologists. As shown in Figures 2 and 3, the histopathologic examination revealed a huge mass in the left lung with necrosis and suppurative caseation. Microscopic examination of caseous material was negative for AFB. PAS staining was positive and cytological examination revealed yeast cells and capsules of non-viable fungi, suggestive of *H. capsulatum*. Because of resource limitations at our hospital, no further tests could be performed. The pathologist's impression was that the patient had had pulmonary histoplasmosis.

## Discussion

In this report, we describe the case of a 15-year-old immune-competent patient who presented to our hospital with chronic productive cough, fever, and massive unilateral consolidation. This case illustrates the many challenges that clinicians face in diagnosing pulmonary histoplasmosis among patients who present to resource-limited health facilities.

The endemic fungi which are primarily human pathogens and whose major portal of entry is the respiratory tract include *H. capsulatum, Blastomyces dermatitidis*, and *Coccidioides immitis *[3]. Histoplasmosis has been shown to be a benign, self-limiting infection in most cases [9], but fatal cases have been reported [10]. The pathogenesis of African histoplasmosis remains unclear. The main route of acquisition could be airborne contamination from the soil and, rarely, direct inoculation [8].

The mycelial form of *H. capsulatum *is found in the soil, especially in areas contaminated with bird or bat droppings, which provide added nutrients for the growth of bacillus. Infections in endemic areas are typically caused by wind-borne spores emanating from point sources such as bird roosts, old houses or barns, or activities involving disruption of the soil, such as farming and excavation [11]. In our case, despite our patient's immune-competent status, rock cutting likely led to a very high level of exposure to *Histoplasma *spores that multiplied easily in his lungs, which had previously been injured by TB.

Despite our patient's different presentation from what has been commonly reported among immune-competent patients, it has already been shown that chronic and severe pulmonary histoplasmosis is associated with pre-existing abnormal lung architecture. Emphysema, for example, has been shown to be a major risk factor for pulmonary histoplasmosis [4]. As in our patient, the symptoms of malaise, productive cough, fever, and night sweats in pulmonary histoplasmosis are similar to those of TB but are usually less severe [4]. Our case therefore illustrates how TB can be a risk factor for pulmonary histoplasmosis and how, in TB-endemic areas, pulmonary histoplasmosis can easily be misdiagnosed as recurrent TB.

Culture remains the gold standard for the diagnosis of histoplasmosis, but it requires a lengthy incubation period (two to four weeks) [4]. Fungal staining produces quicker results than culture but is less sensitive [4]. It has been shown that antigen detection is a sensitive method for diagnosing histoplasmosis, especially in patients with more diffuse pulmonary involvement and those with progressive disseminated disease. Antigen test results vary considerably according to the type of chronic pulmonary disease, with sensitivity ranging from 0% for mediastinal disease, 15% to 21% for chronic pulmonary disease, and 92% for disseminated disease [12-14]. With regard to specimens collected for the diagnosis of histoplasmosis using antigen detection, urine has greater sensitivity than other fluids in the diagnosis of disseminated histoplasmosis; however, optimal diagnostic yield is the result of testing both urine and serum [15]. Histopathological examination, as in our case, typically reveals caseating granulomas whose center, contrary to TB, does not contain AFB but does contain yeast cells or the capsules of non-viable fungi [8,16]. However, the histopathological examination lacks sensitivity and specificity.

One major limitation in our case was that no further tests could be performed to confirm the identification of the fungal elements seen on histopathologic examinations for fungal infection because of the limited diagnostic facilities at our center. As the structure of the *H. capsulatum *yeasts is similar to that of other pathogens, such as *Penicillium marneffei*, *Pneumocystis *(*carinii*) *jeroveci*, *Toxoplasma gondii*, *Leishmania donovani*, and *Cryptococcus neoformans*, misidentification is possible [1]. Limited facilities for fungal identification are a challenge common to many hospitals in resource-limited settings.

Treatment of African histoplasmosis has been discussed elsewhere [8] and can be extrapolated from the guidelines of the Infectious Diseases Society of America established for histoplasmosis due to *H. capsulatum *var. *duboisii *[5]. Treatment is indicated in all patients with chronic pulmonary histoplasmosis. Medications which can be used include amphotericin B, especially in patients with more severe manifestations who require ventilatory support [5]. Ketoconazole and itraconazole can be used but have high relapse rates, with a one-year relapse rate of 95.3% having been reported [5]. Treatment with fluconazole 200 mg to 400 mg daily appears to be even less effective than ketoconazole and itraconazole [5]. It has also been shown that the inflammatory response may contribute to the pathogenesis of respiratory compromise, thus prednisolone 60 mg/day for two weeks can be helpful [17]. Mortality associated with severe histoplasmosis without treatment is 80% but can be reduced to < 25% with anti-fungal therapy [5].

## Conclusion

Although more common in immune-compromised patients, pulmonary histoplasmosis should be considered in the differential diagnosis of immune-competent patients with risk factors including heavy soil exposure and pre-existing lung disease. The presenting symptoms can mimic pulmonary TB. Early diagnosis and treatment are important to improve outcomes.

## Consent

Written informed consent was obtained from the patient's next-of-kin to do an autopsy and publish this case report and any accompanying images. The WBUCHS/BMC ethics review board provided the approval to publish this case report and any accompanying images. A copy of the written consent is available for review by the Editor-in-Chief of this journal.

## Competing interests

The authors declare that they have no competing interests.

## Authors' contributions

RK, SK, RNP, and HJ managed the patient and collected all clinical information. PR and KJ performed post-mortem and histological analyses. SEM performed microbiological analysis and wrote the manuscript. All authors read, edited, and approved the final manuscript.
